# Digital Infrastructure for Antimicrobial Susceptibility Testing and Surveillance: A CLSI and EUCAST-Based Model for Resource-Limited Settings

**DOI:** 10.2196/82727

**Published:** 2026-01-21

**Authors:** Djibril Mbarushimana, Taofeek Tope Adegboyega, Gatera Jean Damascene, Muritala Issa Bale, Buregeya Jean Damascene, Kayitesi Marie Francoise, Itangishaka Innocent, Rugamba Alexis, Rasheed Omotayo Adeyemo, Bagirinshuti Issa, Saheed Adekunle Akinola, Ahmed Adebowale Adedeji, Mushuru Evariste, Busumbigabo Albert, Mukamana Felicite, Habarurema Sylvain, Felix Habarugira, Jean Paul Sinumvayo, Rutambika Noel, Twagirumugabe Theogene, Ndoli Minega Jules, Ngarambe Christian

**Affiliations:** 1 University Teaching Hospital of Butare, P.O. Box 254, Hospital Avenue, Mamba Ngoma, Huye, Rwanda, 250 788575656; 2 Department of Microbiology and Parasitology, School of Medicine and Pharmacy, University of Rwanda, KK 737 Street, Gikondo, Kigali, Rwanda

**Keywords:** antimicrobial susceptibility testing, CLSI, EUCAST, Laboratory Information System, antimicrobial stewardship, resource-limited settings, openClinic GA, University Teaching Hospital of Butare, WHONet

## Abstract

**Background:**

Antimicrobial resistance (AMR) poses a significant global health threat, requiring effective antimicrobial susceptibility testing (AST) and surveillance systems. At the University Teaching Hospital of Butare (CHUB) in Rwanda, a baseline Laboratory Assessment of Antibiotic Resistance Testing Capacity (LAARC) identified critical gaps in the Laboratory Information System (LIS), including low capture rates for culture observation (60%) and AST data (25%), no standardization of AST panels (0%), and limited cumulative antibiogram generation (17%). Existing AMR surveillance platforms, such as the Information System for Monitoring Antimicrobial Resistance by the World Health Organization (WHO) Collaborating Center for Surveillance of Resistance to Antimicrobial Agents (WHONET), and the District Health Information System, operate as standalone systems separate from clinical workflows, which limits their real-time clinical utility.

**Objective:**

This study aimed to develop an enhanced, web-based LIS integrated within routine clinical care to improve AST reliability, enable real-time AMR surveillance at CHUB, and provide a scalable model for subnational and national surveillance networks in resource-limited settings, supporting antimicrobial stewardship.

**Methods:**

We developed an enhanced LIS using the OpenClinic GA, the current open-source hospital information system at CHUB, integrating Clinical and Laboratory Standards Institute (CLSI) and European Committee on Antimicrobial Susceptibility Testing (EUCAST) guidelines, and leveraging metadata from the AMR for R package, WHONET resources, and EUCAST Expert Rules. An agile development approach was used, incorporating a custom database schema, Java-based application programming interfaces (APIs), and web-based user interfaces. The system was designed to support minimum inhibitory concentration (MIC) and disk diffusion (DD) methods, automate result interpretation with color-coded outputs, WHO Access, Watch, Reserve (AWaRe)–based cascade reporting, and enable data export to WHONET for global surveillance.

**Results:**

The enhanced LIS improved AST data capture and standardization, providing reliable, automated result interpretation and real-time AMR surveillance capabilities. The system’s web-based architecture enables scalability through centralized deployment, allowing multiple facilities simultaneous access. Unlike standalone surveillance tools, the enhanced LIS integrates AST within electronic medical records, maintaining clinical information continuity from specimen registration through result reporting. The system supports immediate clinical decision through AWaRe–based cascade reporting, and automated resistance phenotype detection, followed by standardized WHONET-compatible exports for public health surveillance.

**Conclusions:**

This scalable, LIS model demonstrates the feasibility of implementing standards-based AMR informatics in resource-limited settings. By embedding surveillance within clinical workflows rather than treating it as a separate downstream activity, the system maximizes data quality and clinical relevance while minimizing staff burden. The centralized web-based architecture provides inherent scalability from facility to national levels, eliminating data fragmentation and ensuring metadata consistency across networks. Long-term sustainability requires continuous user training, designated personnel for metadata maintenance, local IT capacity building, and funding mechanisms beyond donor dependency. This model provides a practical roadmap for national digital stewardship programs, supporting both immediate patient care and long-term public health surveillance goals.

## Introduction

### Antimicrobial Resistance (AMR) Surveillance Challenges in Resource-Limited Settings

Antimicrobial Resistance (AMR) is a global health emergency, with the World Health Organization (WHO) estimating 10 million annual deaths by 2050 if unchecked [1]. Effective antimicrobial susceptibility testing (AST) and robust surveillance systems are fundamental to combating AMR. AST guides appropriate therapy at the patient level, while surveillance aggregates resistance data to monitor trends and inform evidence-based treatment guidelines [2,3]. Resource-limited settings, particularly in Sub-Saharan Africa, face significant barriers to implementing reliable AST and surveillance infrastructure, including inadequate laboratory information systems, inconsistent adoption of standardized guidelines (Clinical and Laboratory Standards Institute [CLSI] and European Committee on Antimicrobial Susceptibility Testing [EUCAST]),[4] limited access to quality-assured testing protocols, and fragmented data management that impairs interoperability with national and global surveillance networks [5-8], undermine antimicrobial stewardship efforts and exacerbate AMR.

### Baseline Deficiencies at the University Teaching Hospital of Butare

The University Teaching Hospital of Butare (CHUB), a tertiary referral center in Rwanda, exemplifies these challenges. CHUB’s LIS (Laboratory Information System), built on the OpenClinic GA open-source hospital information system [9], exhibited critical deficiencies in microbiology services. A baseline Laboratory Assessment of Antibiotic Resistance Testing Capacity (LAARC) [10], identified critical deficiencies: 60% capture rate for culture observation, 25% for AST data, 0% standardization of AST panels, and 17% generation of cumulative antibiograms, reflecting systemic issues in resource-limited settings. The assessment revealed several qualitative deficiencies in the LIS that compromised bacteriology services at CHUB (Table 1) [11], which reflect systemic issues, where LIS platforms lack specialized microbiology functionality necessary for standards-compliant AST and surveillance.

**Table 1.** Qualitative LIS^a^ deficiencies identified by the LAARC baseline assessment.

Deficiency	Impact
Does not record AST^b^ method: Minimum Inhibitory Concentration (MIC) or Disk Diffusion (DD)	Hinders traceability of test methods
Cannot hide individual antibiotic results	Prevents cascade/selective reporting [12]
Only Susceptible, Intermediate, or Resistant (S/I/R) interpretations entered	Limits data granularity for analysis
Lacks automated interpretation	Manual processes, increasing error risk
EUCAST^c^ Expert Rules not integrated	Limits clinical decision support
Inconsistent antibiotic panels	Results in data unsuitable for reliable cumulative antibiogram generation
Original results deleted during corrections	Compromises audit trails

a LIS: Laboratory Information System.

b AST: antimicrobial susceptibility testing.

c EUCAST: European Committee on Antimicrobial Susceptibility Testing.

### Study Rationale and Objectives

Addressing the revealed gaps requires an enhanced LIS that integrates international AST standards, automates result interpretation, standardizes testing panels, and enables seamless data export to surveillance networks. This study aimed to develop such a system at CHUB leveraging the OpenClinic GA platform and integrating CLSI and EUCAST guidelines [2,3], incorporating metadata from the AMR for R package [13], and from the Information System for Monitoring Antimicrobial Resistance by World Health Organization (WHO) Collaborating Center for Surveillance of Resistance to Antimicrobial Agents (WHONET) [6,14,15], as well as EUCAST Expert Rules and expected phenotypes [16].

The enhanced LIS was designed to resolve all LAARC-identified deficiencies, standardize AST processes, enable real-time AMR surveillance, and facilitate data interoperability with the WHO Global Antimicrobial Resistance Surveillance System (GLASS) and national platforms. By building on OpenClinic GA’s established infrastructure, the system provides a scalable, open-source model tailored to resource-limited settings, improving data quality, supporting evidence-based antimicrobial stewardship, and contributing to global AMR monitoring efforts. This approach offers a replicable blueprint for tertiary hospitals and reference laboratories in Sub-Saharan Africa and similar contexts, demonstrating how standards-based digital infrastructure can address critical surveillance gaps with minimal resource requirements and sustainable, locally adaptable solutions.

## Methods

### Study Design and Setting

This technical implementation study was conducted at CHUB, a key microbiology center in Rwanda, from January 2024 to June 2025. The study focused on upgrading the CHUB LIS, built on the OpenClinic GA open-source hospital information system [9], to address LAARC-identified deficiencies, leveraging metadata from the AMR for the R package and EUCAST Expert Rules.

### System Development Approach

An agile methodology facilitated iterative development, incorporating feedback from laboratory staff, clinicians, and public health experts. Requirements targeted improved data capture, AST panel standardization, antibiogram generation, and interoperability with global surveillance systems, extending the capabilities of the OpenClinic GA platform. The agile approach was chosen based on its proven effectiveness in developing health information systems in low-resource settings [17,18].

### Data Analysis and Metadata Integration

Metadata from the AMR for R package, including microbial taxonomy, antimicrobial profiles, and clinical breakpoints, were analyzed to standardize AST processes. EUCAST Expert Rules and expected phenotypes guided automated interpretations and dynamic testing panel generation. Nine metadata files, all in tab separated values (TSV) format, were integrated, each mapped to specific database tables within the OpenClinic GA system (Table 2).

**Table 2.** Integrated metadata files for LIS enhancement. The integration of these metadata files was based on their reliability and global compatibility [6,19-21].

File content	Purpose	Key fields
Clinical breakpoints	Stores CLSI/EUCAST^a^^b^ breakpoint data defining S/R thresholds	Guideline, test method, pathogen, antimicrobial, breakpoint values
Microorganisms	Provides taxonomic and characteristic data for microbial identification	Pathogen identifier, kingdom, genus, species, prevalence, clinical codes (SNOMED^c^)
Antimicrobials	Details antimicrobial agents with Access, Watch, Reserve (AWaRe) classifications	Antibiotic name, class, potency, administration routes, standardized codes (WHONET^d^)
Antimicrobial screening rules	Defines screening rules for specific pathogens	Antimicrobial, target pathogen, screening criteria
Testable Antimicrobials	Lists antimicrobials with standardized AST^e^ breakpoints	Group, name, abbreviation, AWaRe classification
Expected Phenotypes	Incorporates EUCAST Expert Rules for expected phenotypes	Antimicrobial, pathogen, phenotype (susceptible/resistant)
Site-Sample Mapping	Maps clinical sites to sample types	Site, specimen type (eg, Respiratory → Sputum)
WHONET Specimen Mapping	Standardizes specimen codes for WHONET interoperability	Local codes, WHONET specimen type codes
AWaRe Classification	Classifies antibiotics into AWaRe categories	Antibiotic name, AWaRe category

a CLSI: Clinical and Laboratory Standards Institute.

b EUCAST: European Committee on Antimicrobial Susceptibility Testing.

c SNOMED: Systematized Nomenclature of Medicine.

d WHONET: World Health Organization (WHO) Collaborating Center for Surveillance of Resistance to Antimicrobial Agents.

e AST: antimicrobial susceptibility testing.

### System Architecture and Functionalities

**Figure 1.**
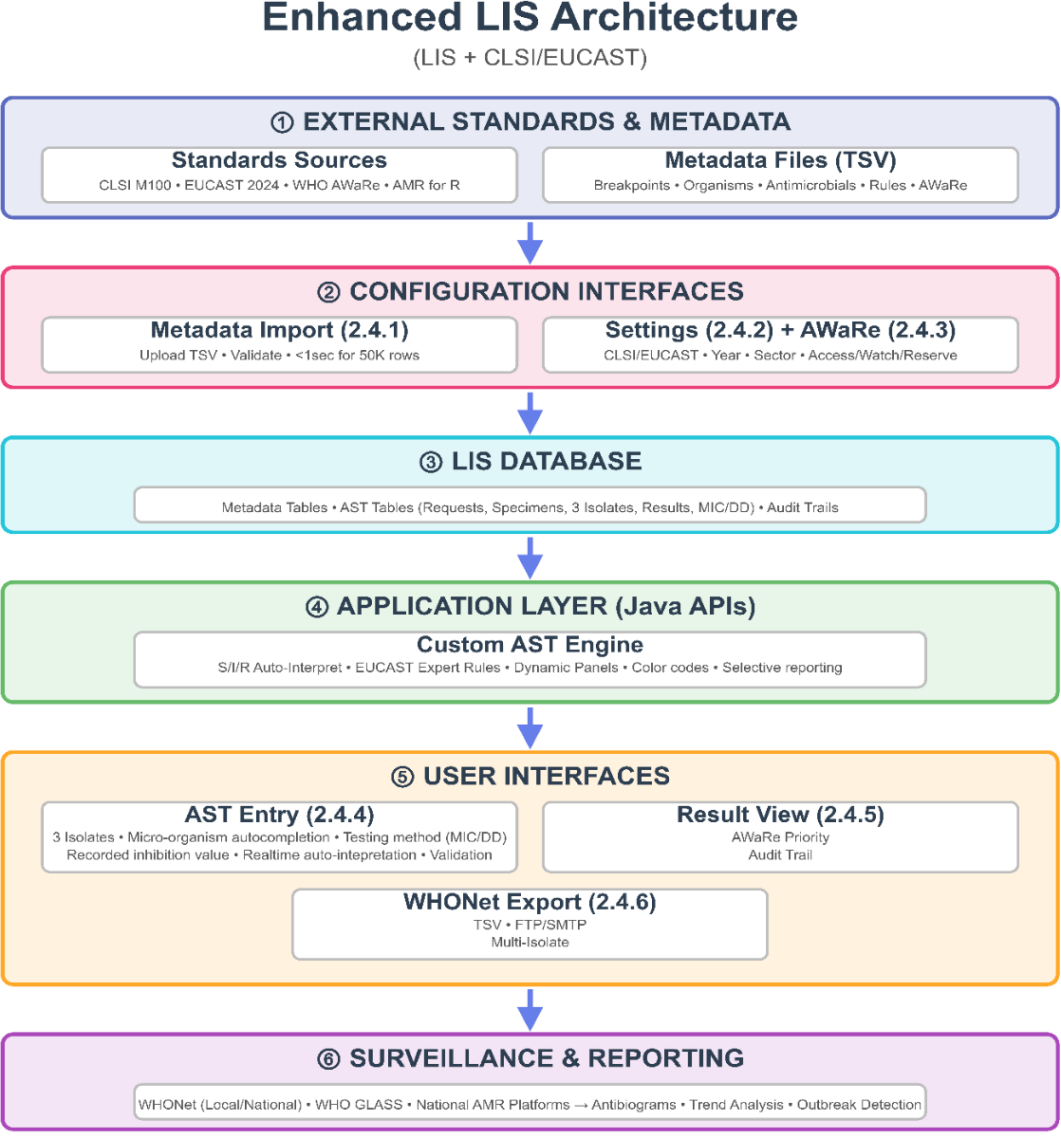
High-level schematic representation of the enhanced LIS architecture and end-to-end data flow. Circled numbers (①-⑥) denote the six architectural layers of the system. Numbers in parentheses refer to subsections in section 2.4 (system architecture and functionalities) where detailed technical descriptions are provided. AST: antimicrobial susceptibility testing; API: application programming interface; AWaRe: Access, Watch, Reserve; CLSI: Clinical and Laboratory Standards Institute; DD: disk diffusion; EUCAST: European Committee on Antimicrobial Susceptibility Testing; LIS: Laboratory Information System; MIC: Minimum Inhibitory Concentration; TSV: Tab Separated Values; WHONET: World Health Organization Collaborating Center for Surveillance of Resistance to Antimicrobial Agents.

The LIS was developed by extending the OpenClinic GA open-source web-based hospital information system, incorporating a custom database schema, Java-based APIs, and a web-based interface, optimized for resource-limited settings. The system comprises five key user interfaces, each designed for usability and compliance with CLSI, EUCAST, and WHO GLASS standards (Figure 1).

### Metadata Import Interface

**Figure 2.**
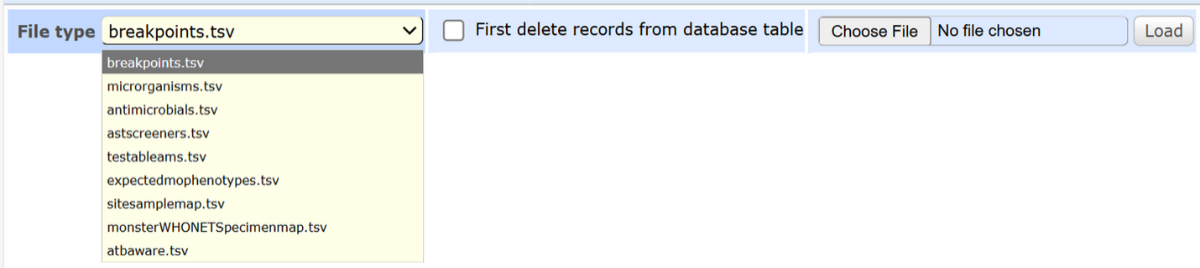
Screenshot of the metadata import interface, showing the dropdown menu for selecting file types, the file upload button, and the feedback panel for validation status.

A web-based tool simplifies the import and update of metadata files within the OpenClinic GA. Users select a file type from a dropdown menu, upload TSV files, and optionally clear existing data. The system validates file headers, provides real-time feedback on errors (eg, invalid format), and deletes temporary files to optimize storage. This interface leverages OpenClinic GA’s robust file-handling capabilities to ensure efficient data management by enabling nontechnical users to update metadata with minimal training (Figure 2).

### Configuration Interface

**Figure 3.**
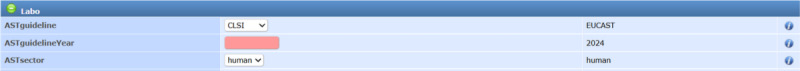
Screenshot of the configuration interface, displaying dropdown menus for selecting AST guideline (CLSI/EUCAST), guideline year, and sector (human/animal), with multilingual support options. AST: antimicrobial susceptibility testing; CLSI: Clinical and Laboratory Standards Institute; EUCAST: European Committee on Antimicrobial Susceptibility Testing.

A web-based tool allows users to configure the AST guideline (EUCAST/CLSI), guideline year, and sector (human or animal) to adapt the system to local needs (Figure 3).

### AWaRe Classification Management Interface

**Figure 4.**
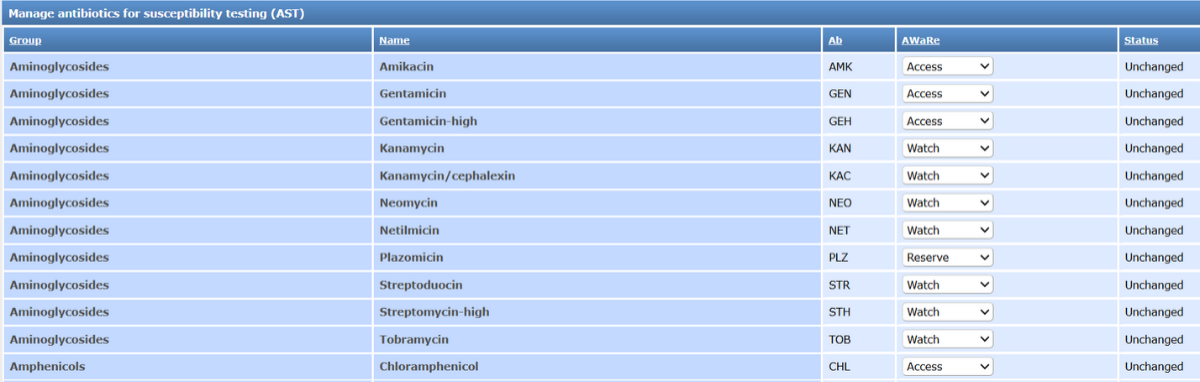
Screenshot of the AWaRe classification management interface, showing a sortable table of antibiotics with dropdown menus for updating AWaRe status and real-time feedback indicators. AST: antimicrobial susceptibility testing; AWaRe: Access, Watch, Reserve.

A dedicated tool manages the AWaRe classification of testable antibiotics. It displays a sortable table of antibiotics with their group, name, abbreviation, and AWaRe status or Not Available. Users update classifications via dropdown menus, with changes tracked client-side using JavaScript and submitted asynchronously to the server for database updates (Figure 4).

### AST Result Entry Interface

**Figure 5.**
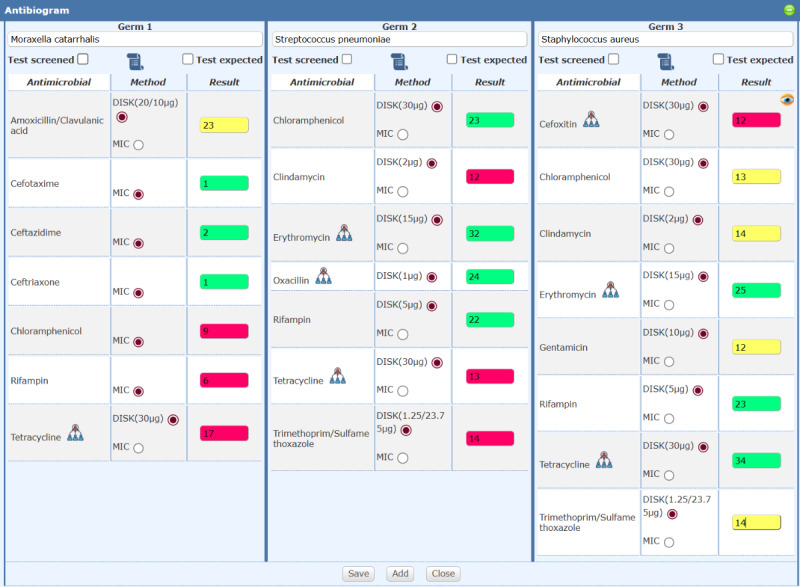
Screenshot of the AST result entry interface, showing the modal dialog with isolate panels, dynamic antimicrobial table, color-coded result fields, and autocompletion for pathogen identification. MIC: minimum inhibitory concentration.

Integrated into worklist management, this modal dialog supports manual entry and validation for up to three isolates per specimen, aligning with CLSI/EUCAST recommendations. Key features include: standardized pathogen identification via WHONET/EUCAST/CLSI taxonomy with autocompletion; dynamic antibiotic panels that expand based on whether to include screened antibiotics and/or those with expected phenotypes; real-time color-coded validation (green=Susceptible, yellow=Intermediate, red=Resistant) for inhibition zones or MIC values; icons indicating related antimicrobials and administration-route-dependent interpretations; alerts for invalid inputs, and incomplete method specification (Figure 5).

### AST Result View Form

**Figure 6.**
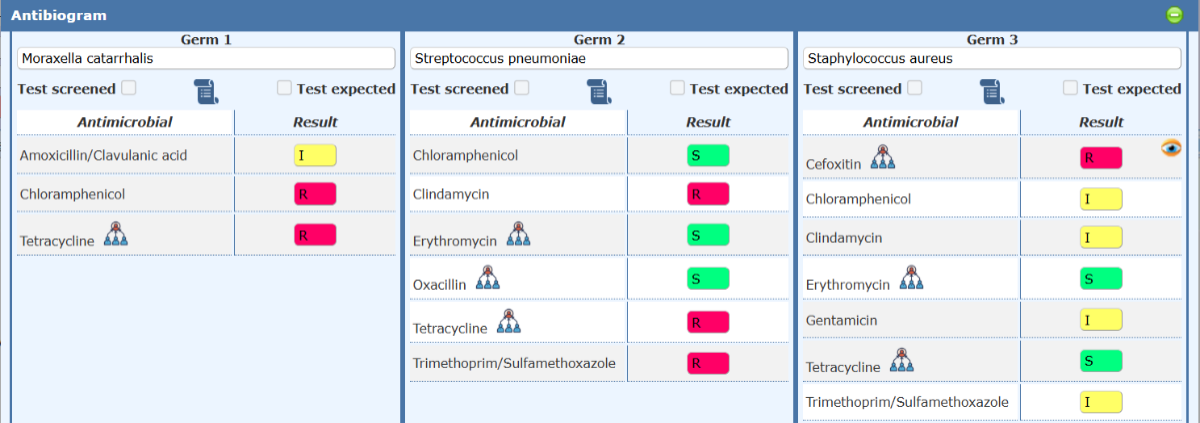
Screenshot of the AST result view form, showing the modal dialog with a grouped results table, color-coded isolate data, AWaRe-filtered antibiotic display, and tooltips for phenotypic comments. AST: antimicrobial susceptibility testing; AWaRe: Access, Watch, Reserve.

Integrated into laboratory results view, this interface displays finalized AST results for clinicians, infection control staff, and surveillance teams, aligning with CLSI, EUCAST guidelines [12,22-24]. Results appear in modal dialogs grouped by patient and request time, showing up to three isolates with organism names, antimicrobials, S/I/R interpretations, and phenotype indicators. AWaRe–based selective display prioritizes Access antibiotics when susceptible or intermediate, then Watch, then Reserve. Read-only format ensures data integrity; edit history icons track corrections with timestamps and user details. Any pathogen isolated triggers red danger icons in the laboratory results overview that corresponds to automated SMS notifications at result validation sent to the patient informing result availability and to the requesting clinician alerting to urgently review results and take clinical action (Figure 6).

### WHONET Export Interface

**Figure 7.**
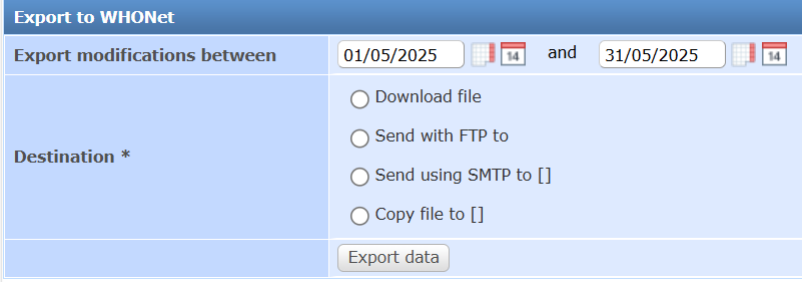
Screenshot of the WHONET export interface, displaying the web form with calendar widgets for date range selection, a dropdown for export destination, and feedback on export status. WHONET: World Health Organization Collaborating Center for Surveillance of Resistance to Antimicrobial Agents.

This interface enables seamless export of AST results to WHONET for global AMR surveillance, complying with WHONET’s data formatting requirements. A web form with calendar widgets allows date range selection and export destination choice (download, FTP, SMTP, or directory). The system queries finalized AST records within the selected range, supporting multi-isolate reporting with patient demographics (ID, sex, age, WHO/CLSI age categories), specimen type (WHONET-mapped), pathogen identification, test method, antimicrobial codes, result values, S/I/R interpretations, guideline, sector, and workflow timestamps. Output generates TSV files matching WHONET import specifications, with legacy CSV support (Figure 7) [25,26].

### Ease of Integration and Interoperability

The enhanced LIS incorporates interoperability features tailored for resource-limited settings, supporting structured data formats (TSV, CSV, JSON, HL7, FHIR) for direct mapping of organism codes, test results, and interpretations. Plug-and-play API endpoints enable the LIS to integrate with other systems with minimal development effort. Direct export to WHONET, WHO GLASS, and national platforms includes automated local-to-surveillance code mapping.

### User Verification, Training and Deployment

Structured user verification and training were conducted before hospital-wide go-live. A dedicated User Acceptance testing was carried out by a team composed of the Laboratory Quality Manager, Microbiology Section Lead, and Information Communication Technology (ICT) staff under the supervision of the Head of Pathology. Nineteen detailed test cases covering system configuration, metadata import, AST result entry (disk diffusion and MIC), dynamic panel generation, EUCAST Expert Rules application, AWaRe–based cascade reporting, audit trails, WHONET export, data integrity, performance, and multilingual support were executed on 10 prospective samples, as per the laboratory standard operating procedure on user verification of LIS. All 19 test cases passed (100 % pass rate). Minor adjustments, mainly related to character encoding compatibility, were applied iteratively.

The six bacteriology laboratory staff received a full day of hands-on training. A 30-minute hospital-wide flash presentation of the new AST reporting features was delivered during clinical grand rounds, and finally, a 5-minute video tutorial covering the entire workflow was produced and disseminated via the hospital’s official WhatsApp groups

The enhanced AST module fully replaced the legacy entry workflow for all new microbiology requests. For backward compatibility and data integrity, the previous (legacy) AST entry and viewing functionality was not removed or disabled. All AST results entered before enhancement remain fully accessible, searchable, and viewable in their original format without any modification or migration issues.

### Ethical Considerations

The project received ethical approval from the CHUB Research-Ethics Committee (REC/UTHB/91/2023 renew, October 20th, 2024); individual informed consent was waived as the study was a quality-improvement intervention using routine clinical data. Patient confidentiality and data protection fully comply with hospital policy through role-based user authentication, display of patient identifiers and clinical information only to authorized clinical users, secure hosting within the hospital’s firewalled data center with twice daily backups, and complete removal of identifiers from all shared data and aggregate datasets.

## Results

The enhanced LIS, built on OpenClinic GA, significantly improved CHUB’s bacteriology services, addressing LAARC-identified deficiencies (Multimedia Appendix 1). The system resolved qualitative gaps identified in the baseline LAARC audit, as detailed below.

### Resolution of Qualitative Deficiencies

The enhanced LIS addressed the qualitative deficiencies identified in the LAARC audit (Section 1.4), all fully resolved. The following Table 3 summarizes the resolution status, referencing relevant system functionalities:

**Table 3.** Resolution status of LAARC qualitative deficiencies.

LAARC deficiency	Status	Evidence and notes
Does not record AST method	Fully Resolved	Captures MIC/DD methods in the AST Result Entry Interface (Section 2.4.4) and includes them in WHONET exports (Section 2.4.5).
Cannot suppress antibiotic results for cascade reporting	Fully Resolved	AST Result View Form (Section 2.4.6) implements AWaRe-based cascade reporting.
Only S/I/R entered, not inhibition zone	Fully Resolved	AST Result Entry Interface (Section 2.4.4) captures inhibition zones/MIC values with automated S/I/R interpretation.
Cannot automatically interpret zone sizes	Fully Resolved	Automates S/I/R interpretation using CLSI/EUCAST breakpoints (Section 2.4.4).
Expert rules not integrated	Fully Resolved	Integrates EUCAST Expert Rules for phenotypic predictions (Sections 2.3, 2.4.4, 2.4.6).
Inconsistent antibiotic panels	Fully Resolved	Achieves 100% panel standardization (Section 3) via metadata-driven panels (Section 2.3) and AWaRe Classification Management Interface (Section 2.4.3).
Original results deleted during corrections	Fully Resolved	Tracks corrections via edit history icons in the AST Result View Form (Section 2.4.6), logging edited results with timestamps, reason and user details.

a LAARC: Laboratory Assessment of Antibiotic Resistance Testing Capacity.

b AST: antimicrobial susceptibility testing.

c MIC: minimum inhibitory concentration.

d DD: disk diffusion.

e WHONET: World Health Organization (WHO) Collaborating Center for Surveillance of Resistance to Antimicrobial Agents.

f AWaRe: Access, Watch, Reserve.

g S/I/R: Susceptible, Intermediate, or Resistant.

h CLSI: Clinical and Laboratory Standards Institute.

i EUCAST: European Committee on Antimicrobial Susceptibility Testing.

### Key Functionalities

The system offers comprehensive AST functionality supporting both MIC and DD methods with automated interpretation based on the selected guideline. Results are displayed using color-coded outputs: green for Susceptible, yellow for Intermediate, red for Resistant, and pink for out-of-range values. The system provides real-time notifications for critical isolates to ensure timely clinical intervention.

Dynamic testing panels automatically adjust based on breakpoints, local antimicrobial stock availability, and clinical context, incorporating EUCAST Expert Rules and expected phenotypes. The cascade reporting feature prioritizes WHO AWaRe “Access” antibiotics, thereby reducing inappropriate use of “Watch” and “Reserve” categories.

For surveillance purposes, data export aligns with WHONET variable definitions, enabling seamless integration with WHO GLASS and national surveillance systems through robust interoperability features. The web-based interfaces, including the AST Result View Form, require minimal training and incorporate real-time feedback and tooltips to enhance usability for nontechnical staff.

## Discussion

The enhanced LIS, built upon the OpenClinic GA open-source hospital information system, addresses critical gaps in CHUB’s microbiology services, offering a robust, standards-compliant solution for AST and surveillance in resource-limited settings. By integrating CLSI and EUCAST guidelines with metadata from the AMR for R package and EUCAST Expert Rules, the system ensures reliable AST results and real-time surveillance capabilities. The agile development approach, coupled with stakeholder engagement, resulted in a user-friendly system accessible to laboratory staff with minimal technical expertise.

The metadata-driven design, particularly the use of WHO AWaRe classifications and EUCAST Expert Rules, optimizes antimicrobial testing and supports stewardship by prioritizing “Access” antibiotics and flagging critical resistance mechanisms. The WHONET export interface enhances interoperability with global surveillance networks, contributing to AMR monitoring and policy development [1].

### Comparison With Existing AMR Surveillance Platforms

The enhanced LIS offers distinct advantages over other popular AMR surveillance platforms by providing comprehensive, integrated laboratory workflow management within a clinical care context. Unlike WHONET, which operates as a standalone desktop on Windows environments and focuses exclusively on bacteriology data [27,28], the enhanced LIS is fully web-based, supports comprehensive clinical laboratory operations across all departments (hematology, biochemistry, immunology, microbiology) and accessible from any device with a standard browser, enabling real-time data entry, validation, and clinical decision support at the point of care. Integrated within the electronic medical record system, the enhanced LIS maintains continuity of clinical information flow from specimen registration through result reporting, directly supporting antimicrobial stewardship through features such as AWaRe-based cascade reporting, automated resistance phenotype detection via EUCAST Expert Rules, and critical alerts that prompt immediate clinical review. While District Health Information System (DHIS2) excels at aggregate health program reporting across multiple domains [29,30], the enhanced LIS provides specimen-level granularity essential for individual patient management, combining automated CLSI/EUCAST-based AST interpretation and quality-assured data validation within a single platform. This integrated approach ensures data serves dual purposes: immediate clinical utility for patient care and antimicrobial stewardship at the facility level, followed by standardized WHONET-compatible exports for public health surveillance at subnational, national, and global levels [6,15]. By embedding AMR surveillance capabilities within routine clinical workflows rather than treating surveillance as a separate downstream activity, the enhanced LIS maximizes data quality, clinical relevance, and public health impact while minimizing the burden on laboratory staff.

### Scalability to Subnational and National AMR Surveillance Networks

The web-based architecture of the enhanced LIS provides inherent scalability for subnational and national AMR surveillance networks through centralized deployment models. The system can be hosted on centralized servers, enabling simultaneous access by multiple testing facilities through standard web browsers without requiring local software installation or high-specification hardware at peripheral sites. This approach ensures real-time data visibility across the network: facility-level users enter AST results through the web interface, while supervisory-level surveillance teams access aggregated data from the same system. From any supervisory level, the WHONET export interface generates standardized data files compliant with WHO GLASS requirements, enabling seamless reporting to global surveillance networks. The centralized model eliminates data fragmentation common in resource-limited settings, reduces infrastructure costs by avoiding multiple server deployments, and ensures metadata consistency (breakpoints, antimicrobial panels, AWaRe classifications) across all participating facilities.

### Sustainability Considerations

Long-term sustainability of the enhanced LIS requires attention to three critical areas. First, continuous user training programs must be established to maintain laboratory staff competency in system use, AST interpretation, and data quality assurance. Second, metadata updates require sustainable mechanisms, including periodic review of CLSI/EUCAST guideline updates, AMR for R package datasets, WHONET resources and taxonomies, local antimicrobial availability changes, and emerging resistance patterns, with designated personnel responsible for metadata maintenance and validation. Third, ongoing technical support requires a multifaceted approach: capacity building for local information technology staff to handle routine troubleshooting, system upgrades, and eventually instrument integration; and sustainable funding mechanisms beyond donor-dependent models to ensure long-term system functionality and relevance.

### Limitations and Future Directions

This study primarily describes the technical development and the qualitative resolution of LIS deficiencies identified in the baseline LAARC audit. Quantitative evaluation of key performance indicators was intentionally deferred to allow sufficient post-implementation observation time. Additional limitations include dependency on manual metadata updates and import, which may be challenging in resource-limited settings. Ongoing training is required to sustain system adoption and ensure data quality. Future enhancements could include automatic metadata updates, mobile app integration, and machine learning for predictive resistance modeling, which have shown promise in other AMR surveillance systems [23,31,32].

### Conclusion

The enhanced LIS, built on OpenClinic GA and leveraging CLSI, EUCAST, and AMR for R metadata, demonstrates the feasibility of implementing standards-based AMR informatics in resource-limited settings. By significantly improving AST reliability, integrating clinical decision support, and enabling seamless integration with global surveillance networks, this model provides a practical roadmap for national digital stewardship programs. The system’s scalability from facility to national levels, combined with its open-source foundation and standards-compliant architecture, offers a replicable blueprint for addressing AMR challenges, supporting both immediate patient care and long-term public health surveillance goals. Widespread adoption of such standards-based digital surveillance systems could significantly contribute to reducing AMR burden through earlier detection of resistance trends and evidence-based policy interventions.

## Supplementary material

10.2196/82727 Multimedia Appendix 1 Demo of new AST (antimicrobial susceptibility testing) reporting.

## Abbreviations

AMR: antimicrobial resistance
API: application programming interfaces
AST: antimicrobial susceptibility testing
AWaRe: Access, Watch, Reserve
CHUB: University Teaching Hospital of Butare
CLSI: Clinical and Laboratory Standards Institute
DD: disk diffusion
EUCAST: European Committee on Antimicrobial Susceptibility Testing
GLASS: Global Antimicrobial Resistance Surveillance System
LAARC: Laboratory Assessment of Antibiotic Resistance Testing Capacity
LIS: Laboratory Information System
MIC: minimum inhibitory concentration
TSV: tab separated values
WHO: World Health Organization
WHONET: World Health Organization Collaborating Center for Surveillance of Resistance to Antimicrobial Agents
